# *Fusarium* Mycotoxins and Their Modified Forms—Occurrence, Toxicity and Analytical Aspects

**DOI:** 10.3390/toxins18060259

**Published:** 2026-06-05

**Authors:** Sanja Furmeg, Vesna Jaki Tkalec, Manuela Zadravec, Ana Vulić

**Affiliations:** 1 Laboratory for Microbiology and Analytical Chemistry, Croatian Veterinary Institute, Veterinary Institute Križevci, Ulica Ivana Zakmardija Dijankovečkog 12, 48260 Križevci, Croatia; furmeg.vzk@veinst.hr (S.F.); jaki.vzk@veinst.hr (V.J.T.); 2 Laboratory for Animal Feed Microbiology, Croatian Veterinary Institute Zagreb, Savska Cesta 143, 10000 Zagreb, Croatia; zadravec@veinst.hr; 3 Laboratory for Analytical Chemistry, Croatian Veterinary Institute Zagreb, Savska Cesta 143, 10000 Zagreb, Croatia

**Keywords:** analytical methods, deoxynivalenol, modified mycotoxins, toxicity, zearalenone

## Abstract

*Fusarium* mycotoxins pose a major challenge for agriculture and the food industry due to their frequent occurrence in cereals. In addition to conventional mycotoxins, modified mycotoxins, including the subgroup of masked mycotoxins, are receiving increasing attention. These compounds are formed through plant defence mechanisms, food processing or biological transformations and are often undetectable using conventional analytical methods. Due to their potential reactivation in the digestive system of humans and animals, masked mycotoxins represent a hidden threat to food safety. This article examines the mechanisms of formation of modified mycotoxins, their occurrence in the food chain and their potential health risks. Particular emphasis is placed on the analytical methods required for their detection, including advanced chromatographic and spectrometric techniques. Understanding modified mycotoxins is crucial for the development of more effective control and prevention strategies. Improved agronomic practices, proper storage and advances in detection methods are essential to reduce exposure to these compounds and ensure food safety. This study provides a comprehensive overview of the current state of research on modified mycotoxins and underlines the need for further scientific research and regulatory guidance to protect consumer health and maintain confidence in the food industry.

## 1. Introduction

Mycotoxins are secondary products of mould metabolism that frequently contaminate food and feed. Cereals, which are widely used in human and animal nutrition, represent the main source of mycotoxins. In agricultural production, contamination of crops with mycotoxins can lead to significant economic losses. In addition to yield losses, additional costs arise from the removal of contaminated food and the implementation of preventive measures to avoid further contamination. The occurrence of mycotoxins and the degree of contamination depend on several physicochemical and biological factors, such as relative humidity, temperature, oxygen availability, the presence of insects, and physical damage to the crop. Mycotoxins can occur in food and feed at any stage of the production process if conditions are favourable [1]. They can be formed on plants during the growing season and harvest, as well as during storage and technological processes [2]. Good agricultural practices are essential to reduce mycotoxin contamination in cereals. Preventive measures should be applied at all stages of production, including soil preparation, crop rotation, hybrid selection, planting date, sowing density, weed control, irrigation, balanced fertilisation, insecticide and fungicide treatment, and harvesting. These measures contribute to systematic and effective contamination control [3]. *Fusarium* species are the most important mycotoxigenic moulds that can contaminate cereals and cereal products [4], such as wheat, oats, barley and maize [5,6]. The most studied mycotoxins include aflatoxins produced by moulds of the genus *Aspergillus*, ochratoxin A produced by *Aspergillus* and *Penicillium*, and the trichothecenes produced by the genus *Fusarium* (type A: HT-2 and T-2 toxin; type B: deoxynivalenol (DON); zearalenone (ZEN); fumonisin B1 (FB1) and fumonisin B2 (FB2)) [7]. Due to extensive research and the considerable risk to human and animal health, the European Union has established maximum levels (MLs) or recommended concentrations for these mycotoxins.

Excessive intake of *Fusarium* mycotoxins can cause acute or chronic mycotoxicosis. Depending on the dose and duration of exposure, genotoxic, nephrotoxic, cytotoxic, oestrogenic and teratogenic effects have been reported in humans and animals [8]. In plants, these metabolites primarily affect seed quality, germination, viability, seedling vigour, root growth and coleoptile [9].

The classification of mycotoxins is challenging due to their chemical structure and biosynthetic origin, their diverse biological effects, and their production by a large number of fungal species [10]. *Fusarium* mycotoxins can be categorised into three classes: trichothecenes, fumonisins, and zearalenone and its metabolites. There are more than 200 different mycotoxins in the trichothecene group, which share a common core structure consisting of an olefinic group, an epoxide group, and a variable number of hydroxyl and acetyl groups [11]. In contrast to trichothecenes, fumonisins are structurally distinct mycotoxins that belong to a group of long-chain aliphatic compounds. Their chemical structure is characterised by a linear carbon backbone with a terminal amino group and two tricarboxylic acid side chains, which confer high polarity and water solubility. The most important representatives are fumonisin B_1_ (FB_1_) and fumonisin B_2_ (FB_2_), which differ in the degree of hydroxylation of the molecule [12]. Zearalenone is a structurally distinct mycotoxin belonging to the group of resorcylic acid lactones. Its chemical structure consists of a macrocyclic lactone ring linked to an aromatic (resorcinol) system, which is responsible for its pronounced oestrogenic activity. Unlike trichothecenes and fumonisins, zearalenone does not contain an epoxide group but possesses phenolic hydroxyl groups that enable various metabolic transformations, including reduction and conjugation [13].

**Figure 1:**
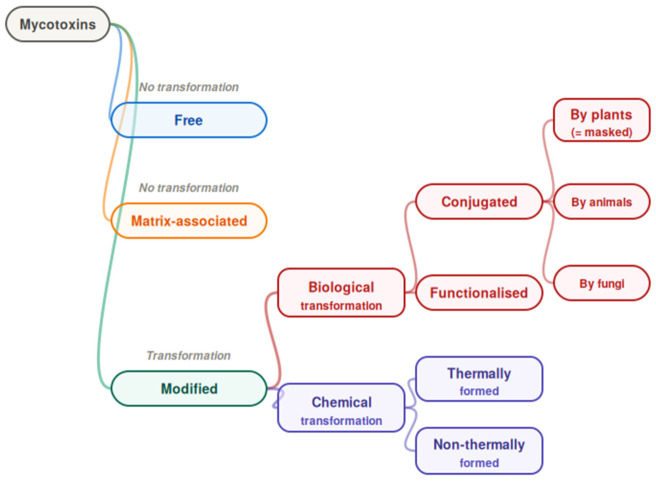
Structural categorization of mycotoxins (according to [17]).

In addition to producing parent mycotoxins, fungi form these secondary metabolites in response to unfavourable environmental conditions, such as stress during crop growth. Once formed, mycotoxins can undergo further transformations in plants, animals, and microorganisms, leading to structurally altered derivatives known as modified mycotoxins. Modified mycotoxins comprise a broad group of compounds that differ from their parent forms due to biological or chemical modifications occurring during plant metabolism, food processing, or digestion. Within this group, masked mycotoxins form a specific subgroup, primarily produced in plants as part of their defence mechanisms against fungal infection. These compounds are typically conjugated with polar molecules, such as glucose, resulting in increased polarity and reduced detectability. Although masked mycotoxins differ structurally from their parent compounds, they may be hydrolysed during digestion, releasing the original toxin and thereby contributing to overall exposure [14]. Their significance was first recognised in the early 1990s, when it became evident that they could not be detected using conventional analytical methods [15]. Despite often exhibiting lower apparent toxicity, masked mycotoxins represent a hidden risk in the food chain due to their potential reconversion into toxic parent compounds [16]. In recent years, numerous previously unknown mycotoxin derivatives have been identified, prompting the development of various classification approaches. One of the most widely accepted classification systems was proposed by Rychlik et al. [17] and is presented in Figure 1.

Group B trichothecenes (DON and nivalenol (NIV)) and zearalenone (ZEN) are the main mycotoxins produced by *Fusarium* species. Food and feed can be contaminated not only with *Fusarium* mycotoxins but also with their numerous metabolites, including modified mycotoxins produced by moulds, plants, animals, and bacteria. The most important fungal metabolites of DON are the acetylated compounds 3-acetyl-DON (3-AcDON) and 15-acetyl-DON (15-AcDON), as well as DON-glucoside (DON-3-Glc), which has been detected in cereals, such as wheat, maize, oats, and barley [18,19,20]. Common modified forms of ZEN include α- and β-zearalenol (α-ZOL and β-ZOL), α- and β-zearalanol (α-ZAL and β-ZAL), zearalenone-14-sulphate (ZEN-14S), and zearalenone-14-O-β-glucoside (ZEN-14G), which occur in plants, moulds, and animals [21]. Recent studies indicate that research on modified myco-toxins has predominantly focused on DON and its derivatives, whereas considerably less information is available for ZEN and its modified forms [22].

Modified fumonisins comprise a heterogeneous group of derivatives formed through chemical, enzymatic, and physicochemical transformations of parent compounds. The most important forms include hydrolysed and partially hydrolysed fumonisins, matrix-bound forms [matrix-associated forms], and Maillard reaction products generated during food processing. Among the most extensively studied derivatives are fully hydrolysed FB1 (HFB1) and FB2 (HFB2), partially hydrolysed forms (pHFB1 and pHFB2), N-(carboxymethyl)-fumonisin B1 (NCM-FB1), and N-(deoxyglucosyl)-fumonisin B1 [23].

Modified mycotoxins pose a potential risk to human and animal health because they cannot be detected by standard analytical methods and may be reactivated in the body to their toxic parent forms. This can lead to various adverse effects, including damage to the liver, kidneys, and immune system, as well as an increased risk of cancer [22]. Modified forms of fumonisins are the focus of a growing number of scientific studies; however, they remain insufficiently investigated compared with their parent compounds. Although numerous derivatives have been identified, data on their occurrence, toxicity, and biotransformation are fragmented, and analytical methods and reference standards are still under development, indicating the need for further research. Despite increasing scientific interest, modified forms of major *Fusarium* mycotoxins have not yet been systematically incorporated into existing regulatory frameworks, primarily due to the lack of comprehensive toxicological data. Available studies on the occurrence of modified derivatives in food and feed are limited, preventing reliable population-level exposure assessment. Furthermore, the detection and quantification of these metabolites are challenging and require the development of highly sophisticated, sensitive, and selective analytical methods that enable their reliable determination in complex food matrices.

The aim of this review is to critically synthesise current knowledge on the formation, occurrence, toxicological relevance, and analytical determination of modified forms of major *Fusarium* mycotoxins, with particular emphasis on deoxynivalenol, zearalenone, and fumonisins, as well as on strategies for their prevention and detoxification. By integrating evidence from plant, fungal, animal, and food-processing studies, this review seeks to identify key knowledge gaps, methodological limitations and regulatory challenges, and to support the development of more comprehensive exposure assessment, monitoring, and risk management strategies.

## 2. Modified *Fusarium* Mycotoxins

Modified mycotoxins include all chemically altered forms of parent mycotoxins that arise from metabolic processes in plants, chemical changes during technological processing, and the action of microorganisms and animals, resulting in compounds with distinct chemical structures and biological activities [24]. They can form during the technological processing of food and feed, such as fermentation, thermal treatment, or other processing procedures, during which partial degradation, isomerisation, or the formation of new derivatives may occur [2]. Table 1 presents an overview of the most important *Fusarium* mycotoxins and their modified forms, including mechanisms of modification, toxicological relevance, and regulatory status, with the aim of providing a more comprehensive understanding of their role in risk assessment.

In the case of deoxynivalenol (DON), modified forms such as 3-AcDON, 15-AcDON, and DON-3-Glc are included in EFSA’s group-based approach to establishing health-based guidance values. However, EU maximum levels remain set only for the parent DON, which may represent a regulatory gap if official monitoring programmes target exclusively the parent compound. For ZEN, EFSA applies a group tolerable daily intake (TDI) expressed as ZEN equivalents, considering the relative potency of phase I metabolites. Nevertheless, EU maximum levels continue to be defined solely for the parent ZEN in cereals and cereal products [25]. With regard to fumonisins, EFSA has established a group for FB1–FB4, but has not included modified forms, such as hydrolysed and other derivatives, within the group health-based guidance value (HBGV) due to insufficient data, thereby increasing uncertainty in the assessment of total exposure. EU maximum levels are prescribed for the parent fumonisins under Commission Regulation (EU) 2023/915 [26].

### 2.1. Modified Mycotoxins by Plants

Plant defence mechanisms can alter the structure of mycotoxins that occur naturally in plants or during food processing, leading to the formation of masked mycotoxins. This defence mechanism proceeds through a multi-phase detoxification system in plants. Mycotoxin metabolites of plant origin are important contaminants in cereals. Phase I consists mainly of hydrolysis, reduction, and oxidation. In this phase, the compounds produced by these reactions may increase in toxicity. In phase II, conjugation and covalent binding occur due to the action of specific enzymes on compounds from phase I. In phase III, the compounds are stored in plant vacuoles or irreversibly bound to the cell wall, forming matrix-associated forms [2,27]. Currently, conjugated mycotoxins are defined as masked mycotoxins if they are present in soluble form, or as bound mycotoxins if they are incorporated into, bound to, or associated with macromolecules. Masked mycotoxins are generally less toxic than their unmodified forms due to chemical changes that alter the polarity of the molecule and thus its bioavailability [16].

### 2.2. Modified Mycotoxins by Microorganisms and Food Processing

In addition to plant-derived conjugates, mycotoxins can also be transformed by mi-croorganisms and during food processing. These modifications may arise through micro-bial metabolism, fermentation, thermal treatment, alkaline processing, or interactions with food matrix components, resulting in structurally altered derivatives distinct from plant-masked forms [17]. Typical examples include the microbial conversion of deoxynivalenol to de-epoxy-deoxynivalenol (DOM-1), the reduction of zearalenone to α- and β-zearalenol, and the formation of hydrolysed fumonisins during nixtamalisation [28].

Such processes are toxicologically relevant because they may alter bioavailability, detectability, and biological activity, while some modified forms may still contribute to total dietary exposure. However, information on the occurrence and toxicological significance of many microbially and process-derived modified mycotoxins remains limited [22].

## 3. Deoxynivalenol and Its Modified Forms

**Figure 2:**
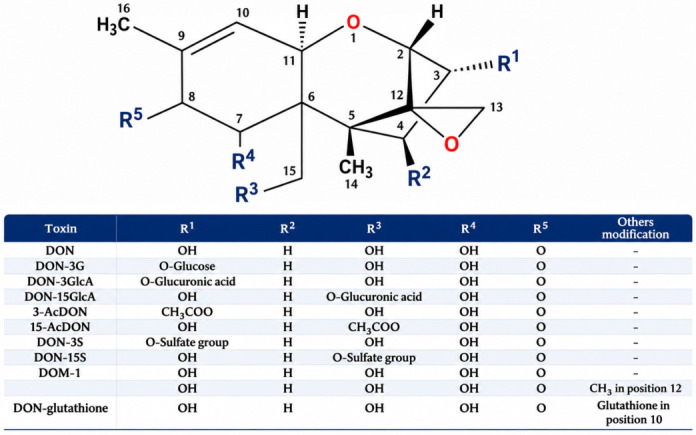
Chemical structure of deoxynivalenol and its modified forms (according to [21]).

DON belongs to the group of B trichothecenes and is a tetracyclic epoxysesquiterpene, also known as vomitoxin. It is one of the most common contaminants in food and feed [29,30]. DON is most frequently found in cereal grains and cereal products, including maize, wheat, barley, oats, and other grains, as well as animal feed, but it can also occur in animal products such as meat and milk. It is produced by the moulds *Fusarium graminearum*, *F. culmorum*, *F. crookwellense*, *F. sporotrichioides*, *F. poae*, *F. tricinctum*, and *F. acuminatum* [31]. Figure 2 shows the chemical structure of deoxynivalenol and its modified forms.

Several forms of mycotoxins have been identified in recent years, among which the most studied masked mycotoxin, DON-3-Glc, is the most abundant plant metabolite of deoxynivalenol and contributes to plant resistance to *Fusarium* infection. The first report on the natural occurrence of DON-3-Glc in wheat and maize contaminated with *Fusarium* spp. was published by Berthiller et al. [32]. To obtain suitable standards, DON-3Glc and deoxynivalenol-15-beta-D-glucopyranoside (DON-15-glucoside) were chemically synthesised. A total of 56 wheat samples were analysed, all of which contained DON and DON-glucoside with the same retention time, molecular weight, and collision-induced dissociation (CID) fragmentation behaviour as synthetic DON-3-Glc. DON-glucoside was also detected in two maize samples naturally contaminated with DON and in five wheat samples, at levels corresponding to 4–12% of the DON concentration. DON concentrations ranged from 4 to 12%. Subsequent studies have confirmed the frequent co-occurrence of DON and its modified forms in cereals and cereal products worldwide. Brodar et al. [33] detected DON and DON-3-Glc in more than half of the analysed cereal samples from the Republic of Croatia. DON is a very common natural contaminant in barley used for beer production and can therefore also be present in beer. Kostelanska et al. [34] conducted a study including 176 beer samples from different producers. The results showed the presence of DON-3-Glc in all samples, with concentrations in some cases even higher than those of free DON. In addition to glucosylated DON, its acetylated forms (AcDON) were also found as contaminants in most of the beers. In general, beers with a higher alcohol content contained higher concentrations of DON and its conjugates. Li et al. [35] analysed maize and wheat samples for the presence of DON and its masked forms. Of the 204 maize samples analysed, DON was found in 50.5% of the samples, while 88 out of 192 wheat samples were positive for DON. DON-3-Glc was detected in maize and wheat samples for the first time in China, with mean levels of 21.4 µg/kg and 34.6 µg/kg for wheat and maize, respectively. Palacios et al. [36] analysed 84 durum wheat samples and found DON in all samples at concentrations ranging from below the limit of quantification (LOQ) (50 μg/kg) up to 9480 μg/kg. DON-3-Glc was detected in 94% of the samples at concentrations from below the LOQ of 50 μg/kg up to 850 μg/kg. Acetylated derivatives were also detected, but with a lower frequency (49%). Klapec et al. [37] investigated the occurrence of deoxynivalenol and its modified forms (3-AcDON, 15-AcDON, and DON-3-Glc) in cereal-based foods from the Croatian market. DON and its derivatives were detected in a wide range of cereal products, with the highest concentrations reported in unprocessed maize (up to 7650 μg/kg) and maize products. Among the analysed food categories, grain milling products showed the highest mean concentration (264 μg/kg), followed by grains intended for direct human consumption (191 μg/kg), pasta (165 μg/kg), breakfast cereals (121 μg/kg), bread and rolls (86 μg/kg), composite foods (55 μg/kg), fine bakery wares (43 μg/kg), and beer (25 μg/kg). In contrast, Olopade et al. [38] reported a low occurrence of DON in cereals from south-western Nigeria, with only acetylated forms detected in a limited number of samples. Such variability can be partly explained by methodological differences among studies, including sampling strategies, climatic conditions, cereal varieties, harvest years, and, most importantly, analytical approaches. Despite numerous studies on DON and its metabolites, data on their occurrence, transformation, and health effects are still incomplete and difficult to compare. Most information is available for wheat and maize, while other cereals such as oats, rye, barley, and triticale are less well studied. A particular challenge is posed by the masked forms of DON, whose presence is often underestimated due to limitations of current analytical methods. Standardised analytical methods, extension of analysis to a wider range of cereals, and additional toxicokinetic studies are needed to assess risks to food safety more accurately. The occurrence of modified mycotoxins can contribute significantly to overall exposure to DON and thus pose a risk to human and animal health; therefore, further studies on masked forms of deoxynivalenol are needed.

## 4. Zearalenone and Its Modified Forms

**Figure 3:**
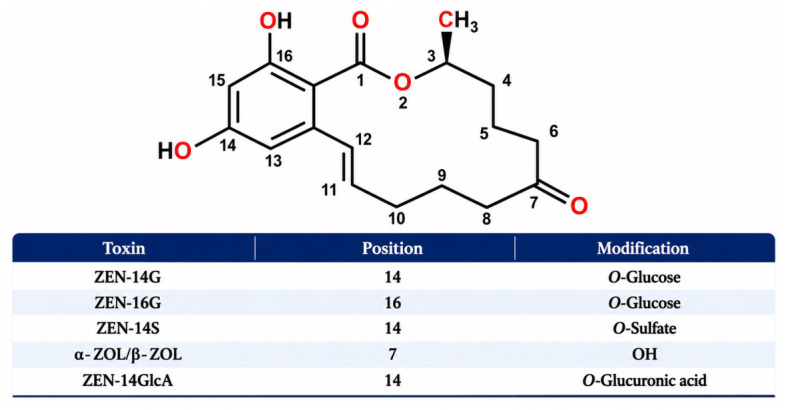
Chemical structure of zearalenone and its modified forms (according to [21]).

ZEN is a mycotoxin belonging to the group of macrocyclic lactones and is also known as F-2 toxin. It is produced by the moulds *F. graminearum*, *F. culmorum*, *F. oxysporum*, *F. equiseti*, *F. verticillioides*, *F. tricinctum* and *F. roseum*. It is classified as an oestrogenic mycotoxin and acts as a phytoestrogen, causing specific oestrogenic symptoms. ZEN has been detected mainly in maize, barley, wheat, rye, soya beans, and their products, but also in milk, muscle, organs, tissues, and eggs of animals fed contaminated feed [31]. Figure 3 shows the chemical structure of zearalenone and its modified forms.

The best known masked forms of zearalenone include ZEN-14G, ZEN-4β-D-glucopyranoside, zearalenone-14-β-D-glucopyranoside (ZEN-14ß-DG), zearalenone-14-β-D-glucopyranoside (ZEN-14ß-DGp), ZEA-2,4-O-β-diglucoside, ZEA-14S, and palmitoyl-ZEA, which are found in wheat bran, corn-based foods (cereal bread, baked goods, breakfast cereals) and vegetable oils [2]. In the study by Brodar et al. [33], 200 cereal samples (maize, wheat, barley, oats, triticale, rye, and spelt) from across the Republic of Croatia were analysed. ZEN was detected in 107 samples, including zearalenone sulphate in 47 samples. In a study by Schneeweis et al. [39], 22 of 24 processed wheat grain samples contained 11–860 µg/kg ZEN, while ZEN-14G (17–104 µg/kg) was found in 10 samples. In a study by Scarpino et al. [40], ZEN was present in 27%, ZEN-14S in 60%, α-ZEL in 5% and β-ZEL in 8% of dry-milled maize samples from north-west Italy. The behaviour of masked zearalenone mycotoxins was described by Gratz [41], who concluded that the masked ZEN compounds ZEN-14G, ZEN-14S and α- and β-ZEL-14G are rapidly hydrolysed by mixed faecal microbiota, with recovery of the intact masked compounds falling below 20–40% after 30 min of incubation. In addition, microbial metabolism of ZEN-14G leads to the complete disappearance of known metabolites (ZEN-14G, ZEN, α- and β-ZEL), which may result in further conversion to as yet unidentified metabolites. These findings indicate that modified forms may contribute to overall exposure through reconversion processes in the gastrointestinal tract, although available data are largely based on in vitro models. Maize cell suspension cultures can convert ZEN, after reduction, into its phase I metabolites, α-ZEL and β-ZEL, and produce glucose conjugates of these compounds, particularly ZEN-14G [18]. According to Berthiller et al. [42], the plant *Arabidopsis thaliana* can convert ZEN and its metabolites into various extractable conjugated compounds, including glucosides, malonylglucosides, dihexosides and pentosylhexosides. The importance of the mould genera such as *Aspergillus* and *Rhizopus* as biotransformers has been noted in the literature. Two strains of *Aspergillus oryzae* and seven species of *Rhizopus* demonstrated the ability to biotransform ZEN into various metabolites, such as ZEN-14S, ZEN-14G, and ZEN-16Glc, as well as phase I metabolites such as α-ZEL and β-ZEL, which also led to the isolation of α-zearalenol sulphate [43,44].

Given the proven toxicity of ZEN and its metabolites, Faisal et al. [45] investigated the interaction between ZEN-14S and cyclodextrins (CDs). Cyclodextrins are cyclic oligosaccharides that can form host–guest complexes with some mycotoxins, including ZEN, zearalenols, and ZEN-14G. Their study showed that ZEN-14S, a modified form of ZEN, can form stable complexes with various cyclodextrins. Dimethyl-β-cyclodextrin (β-CD) showed the highest binding capacity. They also found that ZEN-14S can be successfully removed from aqueous solutions using spherical β-CD polymer. The use of CD technology increases the sensitivity of fluorescence detection of ZEN-14S. In addition, the application of the insoluble CD polymer proved effective for removing mycotoxins and their modified forms from aqueous solutions, including beverages. Further research is needed to evaluate the practical applicability of such approaches in the food industry. The presence of modified forms in substantial proportions in certain matrices underlines their relevance for exposure assessment and monitoring strategies. Although ZEN is one of the most widely studied mycotoxins of the genus *Fusarium*, data on its metabolites, especially masked forms such as ZEN-14G and ZEN-14S, remain insufficient. Most information is available for maize and wheat, while data for other cereals such as oats, rye, and barley are limited. An additional challenge is the complex biotransformation of ZEN in the body and the diversity of its metabolites, whose toxic potential and health effects are not yet fully understood. Therefore, analytical methods for the detection of masked forms need improvement, and more extensive toxicological studies are required to enable comprehensive risk assessment.

## 5. Fumonisins and Their Modified Forms

Fumonisins are a group of mycotoxins produced by certain *Fusarium* species, primarily *Fusarium verticillioides* and *Fusarium proliferatum* [46]. Due to their widespread occurrence, particularly in maize and maize-based products, and their potential health risks, fumonisins are among the most strictly regulated mycotoxins, with clearly defined maximum levels in food and feed [12]. In addition to free forms, modified fumonisins are increasingly reported in food and feed as a result of fungal and plant metabolism or food processing. Although the mechanisms of fumonisin B modification are not yet fully understood, their chemical structure enables interactions with biopolymers in the food matrix. As a result, fumonisins may become physically entrapped within macromolecules such as starch and proteins, forming stable complexes that can be released only after matrix degradation [47]. Furthermore, fumonisins can undergo chemical reactions with food components due to the presence of amino and carboxyl groups, and fatty acid conjugates have been reported in plants and experimentally detected in rice inoculated with *F.* verticillioides [48]. The occurrence of masked fumonisins in raw maize has also been demonstrated, with increased levels of free fumonisins observed after enzymatic digestion, indicating their release from the matrix [49]. This phenomenon is particularly relevant for risk assessment, as modified fumonisins may escape routine detection and contribute to overall exposure. However, compared with other groups, data on the occurrence, toxicokinetics, and toxicological relevance of modified fumonisins remain limited, highlighting the need for further research in this area.

## 6. Modified Mycotoxins: Effects on Human and Animal Health

Modified mycotoxins represent an emerging area of research for which only limited in vivo toxicity data are available. Compared to other trichothecenes, DON is considered one of the less toxic compounds; however, it may have adverse effects on human and animal health after short- or long-term exposure. DON inhibits protein synthesis by binding to ribosomes, leading to the activation of signalling pathways (MAPK) and inducing cell death (apoptosis) in immune and epithelial cells, which can result in immune system dysfunction and increased susceptibility to infections. In humans, DON is associated with gastrointestinal symptoms such as acute nausea, vomiting, diarrhoea, and abdominal pain, as well as possible neurotoxic effects. Several studies have investigated the toxicological properties of the modified form DON-3-Glc. Poppenberger et al. [50] demonstrated a conjugation process for DON-3-Glc that showed a lower ability to inhibit wheat ribosomal protein synthesis in vitro compared to the parent mycotoxin DON. According to the studies of Pierron et al. [51], the glucosylation process of DON suppressed its ability to bind to the ribosome and reduced its intestinal toxicity in vitro in a human intestinal cell line and in vivo on porcine jejunum explants. A previous study by Wu et al. [52] showed that the toxicity of DON-3-Glc is much lower compared with free DON. It has been confirmed that DON-3-Glc has no cytotoxic effect on human gastric epithelial cells [53]. The reconversion of DON-3-Glc to DON and ZEN-14S to ZEN by the intestinal microbiota has been demonstrated [2,54], indicating that modified forms may contribute indirectly to systemic exposure and are therefore relevant for human health risk assessment. ZEN is known as an oestrogenic mycotoxin, as it binds to oestrogen receptors and disrupts hormonal balance. Long-term exposure in humans may cause lasting effects, including menstrual disorders, degenerative changes in the testes, miscarriages, and infertility. Fumonisins are associated with numerous adverse effects on human health, primarily due to their ability to inhibit the enzyme ceramide synthase, which disrupts sphingolipid metabolism and leads to subsequent cellular dysfunction [12]. In view of their classification by the International Agency for Research on Cancer (IARC) as Group 2B carcinogens (possibly carcinogenic to humans), these mycotoxins are among the most strictly regulated. As a result, strict maximum levels have been established for their presence in food and feed, particularly in maize and maize-based products.

Among animals, pigs are the most sensitive to DON exposure. At a DON content of 5 mg/kg in pig feed, feed conversion decreases by 30–50% [55]. As with DON, pigs are also highly sensitive to elevated concentrations of ZEN. Exposure can lead to severe scratching of the tail and back or redness and swelling of the genital area, while chronic exposure, as in humans, may cause permanent effects on the reproductive organs [56]. In contrast, cattle are considered less sensitive because rumen microorganisms are able to degrade ZEN [57]. Ruan et al. [58] were the first to investigate the in vivo toxicity of the masked mycotoxin ZEN-14G and demonstrated despite masked mycotoxins generally being considered less toxic than their parent compounds, ZEN-14G may exert comparable or even enhanced intestinal toxicity under in vivo conditions. The authors showed that ZEN-14G causes GALT dysplasia and significantly altered the intestinal microbiota, bile acid metabolism and the expression of immune proteins in rats. In particular, an increased abundance of *Bifidobacterium* and *Bacteroides* bacteria was observed. These bacteria are involved in the degradation of ZEN-14G and may contribute to intestinal toxicity through the reconversion of the masked form to the parent toxin ZEN. The study also suggests that key intestinal bacteria mediate the toxic effects of ZEN-14G, opening the possibility of developing new models for the study of intestinal nodular lymphoid hyperplasia (INLH). Fumonisins cause a wide range of adverse effects in animals, from subclinical changes in biomarkers and intestinal morphology to clinical syndromes such as pulmonary oedema in pigs and leukoencephalomalacia in horses. Particular attention has been given to modified forms of fumonisins, especially hydrolysed forms such as HFB1 and HFB2 and their N-acylated derivatives. An important aspect of fumonisin toxicology is that, following exposure, toxicity relates not only to the parent compound but also to its biotransformation within the organism. In rats, the in vivo formation of N-acyl-fumonisin B1 has been described; however, its structural similarity to ceramides suggests a further potential for disruption of cellular signalling and membrane integrity. Therefore, risk assessment based solely on parent fumonisins may underestimate the complexity of actual exposure and biological effects [59].

Commission Regulation (EC) No 915/2023 [26] sets the maximum levels of DON in cereals and cereal products for human consumption, while Commission Recommendation 2006/576/EC [60] and Commission Recommendation 2016/1319 [61] provide guidance for animal feed and pet food, respectively. However, current regulatory frameworks primarily address parent *Fusarium* mycotoxins, whereas modified forms are generally not regulated separately, despite their potential contribution to overall exposure in animals.

Despite these findings, many modified mycotoxins identified in in vivo and in vitro studies have not yet been substantiated by comprehensive toxicity data, as general interest in their toxicological properties is still relatively recent. The limited availability and high complexity of in vivo studies represent major obstacles and hinder the collection of information on the health effects of the metabolism of modified mycotoxins. The use of animal studies and the simulation of digestive conditions in vitro facilitates the investigation of the biotransformation of modified mycotoxins resulting from the interaction with gastric acid and the contents of the small intestine, as well as with the intestinal microbiota in the large intestine. Such studies are associated with ethical concerns, as they are conducted on animals. In addition, in vitro studies are very challenging as they involve complex processes of food digestion and biochemical reactions mediated by enzymes and gut microflora in the human gastrointestinal tract [44]. Overall, current evidence indicates that modified mycotoxins, despite their generally lower intrinsic toxicity, may contribute to total exposure through metabolic reconversion and interactions with the intestinal microbiota. Therefore, their toxicological relevance should be considered in future risk assessment and regulatory frameworks.

## 7. Analytical Methods for Detection and Determination

Chromatographic, immunochemical, and electrochemical detection methods, such as liquid chromatography-tandem mass spectrometry (LC-MS/MS), enzyme-linked immunosorbent assay (ELISA), and electrochemical biosensors, are used to detect and quantify mycotoxins and their metabolites [62]. However, changes in the chemical structure of mycotoxins, leading to the formation of masked mycotoxins, render routine analytical methods designed for free mycotoxins unsuitable for detecting modified forms [17]. Liquid chromatography (LC), coupled with various detectors, is a widely used analytical technique for determining mycotoxins in food, feed, and other materials. It offers high sensitivity, resolution, and reliability in mycotoxin detection, making it a widely used and dependable method in laboratory practice. It enables accurate quantitative analysis of mycotoxins, and the systems are highly automated, allowing rapid and efficient analysis of large numbers of samples with minimal labour [63]. The main disadvantages are the high cost of equipment, non-linear calibration curves, carry-over effects, and variations in reproducibility and repeatability [31]. LC-MS/MS is considered the most advanced and widely used technique for analysing mycotoxin traces, as it is more sensitive, specific, and reliable than HPLC [64]. The disadvantages of this method include the high cost of equipment and complex, time-consuming analytical procedures [38]. Liquid chromatography allows separation so that each compound can be quantified individually, while mass spectrometric detectors enable confirmation of the compounds. In addition, new mycotoxin metabolites can be analysed with high-resolution instruments [65,66,67]. Zhang et al. [68] developed a reliable and sensitive ultra-high-performance liquid chromatography (UHPLC) method combined with tandem mass spectrometry (MS/MS) for the simultaneous determination of the mycotoxins ZEN and ZEN-14G in feed, including forage, concentrates, and compound feed. They optimised the sample pretreatment procedure using HLB-SPE cartridges, which improved impurity removal and the enrichment of the target analytes. The method demonstrated satisfactory validation parameters, including linearity, limit of quantification, recovery, and precision, and confirmed the co-occurrence of ZEN and ZEN-14G in feed samples. This method is efficient and suitable for routine monitoring of ZEN and its masked forms in complex food matrices. It contributes to food and feed safety, and its application is recommended for continuous monitoring of mycotoxins in feed mixtures. Zhang et al. [69] developed a method for the accurate quantification of mycotoxins in cereals (maize, wheat, and sorghum) based on isotopically encoded derivatisation. Due to the increasing occurrence of mycotoxins and their various structural forms, there is a constant need to develop and improve analytical methods that can detect and quantify them rapidly, sensitively, and reliably. One important approach is chromatography coupled with targeted high-resolution mass spectrometry (HRMS), which combines liquid chromatography (LC/UHPLC) with HRMS for the targeted detection and quantification of known compounds or mycotoxins. Conversely, chromatography coupled with non-targeted HRMS (also known as non-selective analysis by high-resolution mass spectrometry) enables the detection and identification of a large number of known and unknown compounds in a sample without the need to define the target analytes beforehand. It is used to detect new contaminants [70].

Gas chromatography (GC) is rarely used for mycotoxin analysis, as it often requires a derivatisation step due to the low volatility and high polarity of most mycotoxins [71]. Biosensors are also used for the mycotoxin analysis. They are easy to use, rapid, and sensitive. They enable biological detection and include a physicochemical element that converts this detection into an electrochemical, optical, or thermal signal. Their disadvantages include the need for sample purification and the inability to analyse a large number of analytes simultaneously [72]. In addition to biosensors, immunosensor-based methods are increasingly being investigated, particularly lateral flow immunoassay (LFIA) with Fe-NC monoatomic nanoenzymes, fluorescent meta–organic framework (MOF) biosensor with aggregation-induced emission (AIE) luminogens, and the ratiometric fluorescent ELISA based on AgNCs and calcein–Ce^3+^. Due to the practical limitations of immunochemical methods for mycotoxin detection, such as high costs, limited antibody lifetime, and antibody variability, alternative affinity technologies are being intensively developed. The most promising substitutes for antibodies are aptamers (nucleic acid molecules), molecularly imprinted polymers (MIPs), and short-chain peptides. Due to their robustness, low cost, and suitability for use under field conditions, these alternatives are well suited for the rapid screening of mycotoxins. In recent years, numerous biosensors based on these recognition elements have been developed, with a focus on innovative transduction and signal amplification strategies. These include DNA walkers, isothermal amplification methods, smartphone-assisted optical approaches, and microfluidic and paper-based platforms. Methods of particular interest include multichannel self-powered electrochemical systems and lateral flow assays with nanozymes, which allow multiplex detection and signal amplification. While these systems have demonstrated high sensitivity and specificity, their application to real matrices remains limited due to a lack of validation in complex food samples. A key step to expand the application of these technologies is to perform tests on real samples and under practical conditions, considering the analytical parameters related to the food matrix [70]. Advanced analytical methods are now available that allow the simultaneous detection and quantification of a large number of mycotoxins, including their modified forms. In addition, modern methods are more sensitive, selective, and time-efficient. This technological progress has significantly facilitated the analysis of their presence in different matrices and enabled a better assessment of mycotoxin exposure in humans and animals. The principal characteristics, advantages, and limitations of the most commonly applied analytical approaches for the detection of parent and modified mycotoxins are summarised in Table 2.

Sample preparation is a crucial aspect of mycotoxin analysis, particularly due to the diversity of matrices and the presence of both parent mycotoxins and their modified forms [73]. The most commonly used approach prior to instrumental analysis combines extraction with an organic solvent (a mixture of methanol and/or acetonitrile with water), a dilution step, and subsequent instrumental analysis. The extraction mixture is often adjusted in terms of the ratio of organic to aqueous components to optimise the extraction of compounds with different polarities. Acids are frequently added to the extraction mixture to improve recovery [16]. This approach allows for short sample preparation times, but has the disadvantage that the matrix may interfere with instrumental analysis. In the past, this method resulted in high limits of quantification (LOQ) and detection (LOD), but modern instruments are more sensitive, leading to significantly lower LOQ and LOD values. To obtain a cleaner extract and reduce matrix effects, as well as to concentrate analytes, samples are often subjected to solid-phase extraction (SPE), either using immunoaffinity (IA) columns or various types of sorbents, such as C18, C8, or HLB. A disadvantage of IA columns, apart from longer sample preparation times, is their lower selectivity; these columns can bind the native form of the mycotoxin, but binding of its metabolites is uncertain. Another sample preparation method that has recently gained attention is the QuEChERS (Quick, Easy, Cheap, Effective, Rugged and Safe) method [74]. QuEChERS-based methods offer many advantages, including rapid sample preparation, low cost, and ease of use. In research by Pascari et al. [75], the QuEChERS method was used to prepare oat and wheat flour samples for the analysis of ZEN and its modified forms, including ZEN-14S, α-ZEL-14S, and β-ZEL-14S. Samples were treated with the enzymes α-amylase and amyloglucosidase to monitor the effect of exposure to ZEN-related compounds on these enzymes. An innovative sample preparation method is freezing combined with magnetic purification [76]. Recent research highlights the importance of using appropriate microfilters, as some filters can cause the loss of mycotoxins. Filtration is intended to remove particles that could damage the column or interfere with analysis. The use of a polytetrafluoroethylene (PTFE) filter results in the lowest mycotoxin losses, followed by nylon. However, polyethersulfone (PES), mixed cellulose ester (MCE), and cellulose acetate (CA) should be avoided, as they can significantly reduce recovery, especially for compounds such as ZEN [77]. Researchers have also developed the Python programme FPScreener (Fragmentation Pattern Screener), which enables rapid isolation of different classes of mycotoxins. This protocol allows for the assessment of unknown parent and modified mycotoxins in food matrices and has successfully identified six parent mycotoxins and eight modified mycotoxins (with varying degrees of confidence) in contaminated wheat and flour samples [70,78]. As analytical methods for the detection and quantification of mycotoxins advance, sample preparation methods are also being continuously improved. These improvements aim to increase the sensitivity, selectivity, and accuracy of mycotoxin analysis.

## 8. Prevention of Mycotoxin Occurrence and Detoxification Processes

Given the frequent occurrence of mycotoxins in cereals and cereal products, the implementation of comprehensive control strategies throughout the entire production chain is essential. Effective mycotoxin management relies on a combination of preventive measures before harvest, control strategies after harvest, and detoxification and decontamination procedures aimed at reducing toxin levels once contamination has occurred.

### 8.1. Pre-Harvest Strategies

According to Hojnik et al. [79], preventing mycotoxin occurrence in the field remains the most effective and economical preventive measure. However, mycotoxin occurrence is difficult to avoid due to ongoing and projected climate change, which is expected to lead to increased average annual temperatures, more frequent droughts and more frequent extreme precipitation events, including flooding. Measures to prevent mycotoxins occurrence in the field include crop rotation to reduce pathogen incidence, selecting resistant varieties, optimising sowing and harvesting times, proper fertilisation, and protecting crops with fungicides and insecticides. In addition to conventional agronomic practices, genetic resistance represents an important strategy for mitigating mycotoxin contamination. Scientists have shown that a key mechanism for DON tolerance in barley is glycosylation, a process facilitated by the enzyme uridine diphosphate-dependent glycosyltransferase (UGT). This process produces the less toxic compound DON-3-Glc. The development of transgenic wheat varieties with a barley gene (HvUGT13248) that converts DON into DON-3-Glc has proven effective. These transgenic plants contain lower toxin levels in the flower and are more resistant to diseases caused by *Fusarium* species, reducing damage by up to 50% [80,81].

### 8.2. Post-Harvest Strategies

The application of good technical practices (GTP) after harvest is crucial for reducing mould growth and mycotoxin production. Post-harvest strategies primarily aim to prevent further fungal growth and additional mycotoxin production after harvest. Inadequate handling, drying, and storage conditions may significantly increase the risk of contamination, even when appropriate preventive measures have been applied during cultivation. Therefore, the implementation of good post-harvest practices is essential for maintaining product quality and safety. Key post-harvest measures include rapid and effective drying to reduce moisture content, sorting and removal of damaged or contaminated grains, control of insects and rodents, and maintenance of optimal storage temperature and humidity. Furthermore, proper hygiene of storage facilities, adequate ventilation, and continuous monitoring of environmental conditions contribute to limiting mould development and mycotoxin accumulation [82]. The effectiveness of post-harvest strategies largely depends on their timely application and consistent implementation throughout storage and distribution. An integrated approach combining multiple preventive measures is considered the most efficient way to minimise the risk of mycotoxin contamination during the post-harvest phase.

### 8.3. Detoxification and Decontamination

In addition to the application of good agricultural practices during cereal cultivation, which reduce the occurrence of mycotoxins, post-harvest detoxification measures are increasingly being applied. These approaches aim to reduce the toxicity, bioavailability, or concentration of already formed mycotoxins and thereby minimise their adverse effects on human and animal health. In this context, detoxification strategies can be broadly classified into physical, chemical, biological, and innovative approaches that are still under development. Physical strategies to reduce mycotoxins include sorting, proper and rapid drying, post-harvest insect control, good storage conditions, and the use of inorganic and organic adsorbents such as montmorillonite and yeast cell walls. Feed additives based on mycotoxin adsorbents act primarily by binding toxins in the gastrointestinal tract, reducing their bioavailability and facilitating their excretion as toxin–adsorbent complexes. Inorganic binders such as bentonites, montmorillonite, and hydrated sodium calcium aluminosilicates (HSCAS) have demonstrated high efficacy, particularly against aflatoxins, whereas their effectiveness against other mycotoxins such as deoxynivalenol, ochratoxin A, fumonisins, and T-2 toxin remains limited. Non-specific adsorption may also occur, leading to unintended binding of nutrients and potentially compromising the nutritional value of feed. Therefore, the use of mycotoxin adsorbents is subject to regulatory evaluation, particularly within the European Union, where their safety, efficacy, and selectivity must be demonstrated before approval as feed additives [83].

In addition to these conventional measures, modern oxidation technologies such as irradiation and cold plasma treatment enable the rapid degradation of mycotoxins through structural modification of toxin molecules [82,84]. Chemical approaches include the use of bases such as ammonia and oxide hydrate. However, the European Union has banned this detoxification process for food samples intended for human consumption. Other chemical methods include ozone treatments and the use of chitosan, which inhibits fungi, bacteria and viruses. Alongside physical and chemical interventions, biological detoxification methods have gained increasing attention. These include processes in which bacteria, yeasts, fungi, fermentation, and enzymatic degradation are used to break down mycotoxins. Owing to their high specificity and ability to produce less harmful metabolites, biological approaches are considered promising alternatives. In addition to these methods, innovative techniques such as the use of nanoparticles and plant extracts are being developed to further enhance detoxification efficiency [80]. DON is highly thermostable and can withstand temperatures of 170 to 350 °C, making it difficult to detoxify. Consequently, alternative approaches are required, and detoxification of DON mainly relies on biological methods, phytochemicals and nanoparticles. Increasingly, plant extracts are used to reduce the harmful effects of DON, as they bind the toxin and convert it into less harmful forms. These extracts contain compounds such as polyphenols from green tea and curcumin from turmeric, which act as antioxidants and protect cells. Moreover, some extracts may stimulate enzymes involved in toxin elimination [80,85].

Biological degradation has been extensively investigated for ZEN. Due to their high specificity and ability to produce harmless by-products or even achieve complete detoxification under suitable conditions, biological detoxification methods using fungi and bacteria are increasingly being applied. For example, some fungi, such as *Thamnidium elegans* and *Mucor bainieri*, can convert ZEN into less toxic forms such as ZEN-14G. The fungus *Gliocladium roseum* produces the enzyme lactonase, which cleaves the lactone ring of ZEN and detoxifies the toxin. *Trichosporon mycotoxinivorans* is a fungus with a high capacity to degrade ZEN, producing non-oestrogenic metabolites. In addition, various isolates of the genus *Rhizopus* can completely degrade ZEN, and *Aspergillus niger* can detoxify ZEN by sulphonation. Moreover, many bacteria, including species of the intestinal microbiota as well as *Acinetobacter*, *Pseudomonas*, and *Rhodococcus*, have also demonstrated the ability to degrade ZEN [86].

Masked mycotoxins do not harm the plant in which they occur. However, if ingested by humans or animals, they may cause adverse health effects. Although masked mycotoxins are less toxic, they are likely to be hydrolysed into free forms by intestinal microorganisms in the mammalian digestive tract, contributing to unanticipated toxicity [26]. Studies of ZEN-14G metabolism in liver microsomes of various animal species (rats, chickens, pigs, goats, cows) and humans have revealed that hydrolysis of the masked form occurs via a primary metabolic pathway involving reduction, hydroxylation, and glucuronic acid (GlcA) conjugation [87]. Under in vitro conditions, the human gut microbiota can deconjugate masked mycotoxins DON-3-Glc, ZEN-14G and ZEN-14S, resulting in the release of toxic aglycones and the formation of unspecified catabolites. The parent mycotoxins released by the transformation of masked forms may lead to increased toxic effects in exposed individuals. Therefore, masked mycotoxins must be considered when assessing population exposure to mycotoxins [88,89]. ZEN-14S and DON-3-Glc are the most frequently detected masked mycotoxins in animal feed. Researchers are investigating their toxicological properties, particularly the conversion of DON-3-Glc to DON and ZEN-14S to ZEN by the microbiota in the digestive tract [90,91]. More generally, mycotoxins can be converted into less toxic or non-toxic metabolites by biotransformation, often carried out by living organisms or isolated enzymes. These transformations mainly include hydroxylation, oxidation, hydrogenation, deep oxidation, methylation, glycosylation, glucuronidation, esterification, hydrolysis, sulphation, demethylation, and deamination [92]. While in vitro studies have shown that masked forms have lower toxic effects on animal and human cells than free mycotoxins, in vivo studies have demonstrated that masked forms can be significantly toxic due to their conversion into free forms by enzymatic reactions [93].

## 9. Future Perspectives on Modified Mycotoxins

Modified mycotoxins are an increasingly important topic, as they can significantly impact food safety and agriculture due to their ability to bind to other compounds and be released under certain conditions. Formed through complex biochemical processes in moulds, plants, and animals, modified mycotoxins present a major challenge for detection because of their diverse structures and various forms. These substances are modified or conjugated forms of conventional mycotoxins. However, the potential to revert to the original, more toxic form under specific conditions increases the risk to consumer health. As the occurrence of modified mycotoxins is closely linked to that of the parent mycotoxins, continuous improvement of agricultural practices, storage methods, and the development of mycotoxin-resistant crops is essential to reduce the overall presence of mycotoxins, including modified forms. Their structural complexity and variability make detection particularly challenging. Consequently, they often remain undetected by routine analytical methods for mycotoxins detection, posing a hidden threat to human and animal health, as well as to the global food supply. Despite advances in mycotoxin analysis, our understanding of their effects on animal and human health through food products remains limited. The identification of new forms of contamination with different *Fusarium* mycotoxins necessitates further toxicological studies. Some regulations, particularly those of the European Union, have begun to address the risk of modified mycotoxin contamination in the food chain. Future research on the formation and occurrence of modified mycotoxins, improvements in analytical detection methods, risk assessment strategies, and the effects of modified mycotoxins on human and animal health is crucial from a health perspective.

## 10. Conclusions

*Fusarium* mycotoxins represent a persistent and globally significant food and feed safety issue, and a growing body of evidence indicates that their modified forms may substantially contribute to the overall exposure of humans and animals. Although a large number of derivatives have been identified, particularly modified forms of deoxynivalenol, zearalenone and fumonisins, data on their occurrence, toxicity and biotransformation remain insufficient for reliable risk assessment. The difficult detection and quantification of modified forms in complex matrices represent a major analytical challenge, highlighting the need for sensitive and validated multimethod approaches, primarily based on LC-MS/MS technology. Monitoring systems should focus on priority cereals, especially maize, wheat, barley and oats, as well as products intended for infant nutrition and feed. Despite increasing scientific interest, modified forms have not yet been systematically incorporated into regulatory frameworks due to limited toxicological data and inadequate exposure assessment. Therefore, further multidisciplinary research is required to elucidate the mechanisms of formation, toxicological relevance and biotransformation of these compounds, to develop standardised analytical protocols and to support their gradual inclusion in monitoring and risk management programs.

## Figures and Tables

**Table 1:** *Fusarium* mycotoxins and their modified forms: mechanisms, toxicological relevance and regulatory status.

*Fusarium* Mycotoxin	Modified Forms	Modification Mechanism	Toxicological Relevance	EU Regulatory Status
DON	DON-3-Glc	Plant glucosylation	DON—confirmed toxicity; MODIFIED FORMS—contribution on total DON exposure	Commission Regulation (EU) 2023/915 applies only to DON; there is no separate ML for modified forms
	3-AcDON	Fungal acetylation		
	15-AcDON	Fungal acetylation		
ZEN	ZEN-14-glucoside	Plant glucosylation	ZEN—primary contributor to total oestrogenic exposure MODIFIED FORMS—ZEN—equivalents; data limited	Commission Regulation (EU) 2023/915 applies only to ZEN; there is no separate ML for modified forms
	ZEN-14-sulphate	Plant sulphation		
	α-zearalenol	Enzymatic reduction		
	β-zearalenol	Enzymatic reduction		
Fumonisins (FB1, FB2)	Hydrolysed fumonisins (HFB1, HFB2)	Processing—induced hydrolysis	FB1, FB2—pronounced hepatotoxicity and nephrotoxicity MODIFIED FORMS—limited in vivo toxicological data; lower potency than the parent compound	Commission Regulation (EU) 2023/915 applies only to fumonisins; There is no separate ML for modified forms

**Table 2:** Comparison of analytical techniques for the determination of free and modified mycotoxins.

Analytical Technique	Target Analytes	Advantages	Limitations	Applicability (Free vs. Modified Forms
HPLC	Primarily free (parent) mycotoxins (e.g., FB1, FB2, ZEN, DON)	Robust, well-established methods; widely available instrumentation	Requires derivatisation; limited selectivity; generally unsuitable for most modified forms	Suitable for parent mycotoxins; unsuitable for most masked or hydrolsed forms without prior hydrolysis
LC-MS/MS	Free and known modified forms (e.g., HFB1, HFB2, DON-3Glc, DON-3AcDON, DON-15AcDON	High sensitivity and selectivity; multi-analyte capability; no derivatisation required	Requires certified reference; matrix effects; relatively high instrument cost	Gold standard for simultaneous determination of free and known modified forms
HRMS	Free forms plus unknown or emerging modified forms	Non-target screening; retrospective data analysis; identification of previously unrecognised metabolites	More complex data processing; typically lower quantitative precision than triple quadrupole; expensive instrumentation	Particularly suitable for research applications and the identification of novel modified forms
ELISA	Primarily free mycotoxins	Rapid and simple screening; high sample throughput	Potential cross-reactivity; inability to distinguish structural analogues; limited quantitative reliability	Appropriate for screening of free forms; unreliable for specific detection of modified forms

## Data Availability

No new data were created or analyzed in this study. Data sharing is not applicable to this article.
